# Analysis of electrochemical impedance spectroscopy data for sputtered iridium oxide electrodes

**DOI:** 10.1088/1741-2552/add090

**Published:** 2025-05-07

**Authors:** Henry M Lutz, Yupeng Wu, Cynthia C Eluagu, Stuart F Cogan, Kevin J Otto, Mark E Orazem

**Affiliations:** 1 Department of Chemical Engineering, University of Florida, Gainesville, FL, United States of America; 2 Department of Material Science Engineering, University of Texas at Dallas, Richardson, TX, United States of America; 3 Department of Bioengineering, University of Texas at Dallas, Richardson, TX, United States of America; 4 Affiliate, J. Crayton Pruitt Family Department of Biomedical Engineering, University of Florida, Gainesville, FL, United States of America; 5 Weldon School of Biomedical Engineering, Purdue University, West Lafayette, IN, United States of America

**Keywords:** iridium oxidation state, porous electrodes, measurement model, ohmic impedance

## Abstract

*Objective*. Our objective was to perform a complete analysis of *in-vitro* impedance data for sputtered iridium oxide film (SIROF) micro-electrodes. The analysis included quantification of the stochastic and bias error structure and development of a process model that accounted for the chemistry and physics of the electrode–electrolyte interface. *Approach*. The measurement model program was used to analyze electrochemical impedance spectroscopy (EIS) data for SIROF micro-electrodes at potentials ranging from −0.4 to +0.6 V(Ag|AgCl). The frequency range used for the analysis was that determined to be consistent with the Kramers–Kronig relations. Interpretation of the data was enabled by truncating frequencies at which the ohmic impedance influenced the impedance. *Main results*. An interpretation model was developed that considered the impedance of the bare surface and the contribution of a porous component, based on the de Levie model of porous electrodes. The influence of iridium oxidation state on impedance was included. The proposed model fit all 36 EIS spectra well. The effective capacitance of the SIROF system ranged from 32 mF cm^−2^ at −0.4 V(Ag|AgCl) to a maximum of 93 mF cm^−2^ at 0.2 and 0.4 V(Ag|AgCl). *Significance*. The model developed to interpret the impedance response of neural stimulation electrodes *in vitro* guides model development for *in-vivo* studies.

## 1. Introduction

Micro-electrodes have been evaluated for their ability to provide electrical stimulation to different systems within the body including muscle [1] and brain [2]. Electrical stimulation is conducted as part of probing and therapeutic efforts [3]. The performance of micro-electrodes is controlled by two key parameters: their impedance and their charge-storage capacity [1]. Porous electrode systems are commonly used for their increased effective surface area that increases the density of charge storage [4]. Additional concerns for the implanted electrodes include the impact on the body caused by foreign body responses and the impact of toxic products of electrochemical reactions associated with introduced electrolytes [5]. Electrode systems that are able to use physiological fluids as electrolytes provide an opportunity to avoid the introduction of foreign electrolytes [6]. Efforts have shown that foreign body responses are lowered in impact when using smaller electrodes [7]. Sputtered iridium oxide film (SIROF) micro-electrodes (200–2000 $\mu \mathrm{m}^2$) and ultramicro-electrodes ($ < $200 $\mu \mathrm{m}^2$) are porous electrode systems studied for their ability to provide primarily charging current during stimulation due to their high-charge-storage capacity [8]. SIROF electrodes also have the ability to charge and discharge using bodily fluids as electrolytes [9].

SIROF electrodes have been studied for long-term stability using techniques such as cyclic voltammetry (CV), electrochemical impedance spectroscopy (EIS), voltage transients, and direct current stimulation [10–12]. SIROF ultramicroelectrodes have shown stability for current pulsing of over 10^9^ cycles in phosphate buffered saline [13]. The charge-storage capacity of SIROF electrodes has been studied with both CV and current pulsing *in vitro* with phosphate buffered saline, and the results indicate that SIROF electrodes ranging from 20 to 2000 *µ*m^2^ exhibit suitable electrochemical properties for neural stimulation [7]. Published work with SIROF micro-electrodes has reported a volumetric capacitance of 425 $\mathrm{F}\,\mathrm{cm}^{-3}$ [9].

Pickup and Birss [14] studied the kinetics of the oxidation and reduction of iridium. Conway *et al* [15] suggest that the pseudocapacitance associated with redox processes contribute to the impedance as a parallel combination of a capacitor and resistor. Sziráki and Bóbics [16] studied the impedance response of the Ir(III)/Ir(IV) electrochromic reaction on electrodeposited AIROF electrodes. They proposed that the redox process can appear as a series combination of a resistance, a capacitance, and a Warburg impedance.

The objective of the present work is to apply the measurement model approach pioneered by the Orazem group [17–19] to impedance data collected on SIROF micro-electrodes in phosphate-buffered saline as a function of applied potential. The data were collected in triplicate, allowing assessment of the stochastic error structure. The interpretation model accounts for the porous electrode structure and the influence of the iridium oxidation state transitions on the impedance response.

## 2. Methods

The methods used to deposit the SIROF films, measure impedance, and interpret the resulting spectra are presented in this section.

### 2.1. Experimental

SIROF micro-electrodes were fabricated by thin-film deposition. The details of the fabrication method were previously published [5]. The deposition was performed with a custom-modified DC magnetron sputtering system (AJA ATC 2200) with 100 W DC power on Ir targets, 20 $\mathrm{stp\;cm}^3\mathrm{min}^{-1}$ of Ar, 7.5 $\mathrm{stp\;cm}^3\mathrm{min}^{-1}$ of $\mathrm{O}_2$, and 22.5 $\mathrm{stp\;cm}^3\mathrm{min}^{-1}$ of $\mathrm{H}_2\mathrm{O}$ at 30 mTorr chamber pressure. A thin layer of titanium was deposited as an adhesion layer before the SIROF deposition. The resulting SIROF films had a thickness of 320 nm. The SIROF micro-electrode used to collect the EIS spectra had a geometric surface area of 2000 *µ*m^2^. Potentials were referenced to a Ag|AgCl reference electrode in a three-electrode cell configuration. The electrolyte was Argon-sparged phosphate buffered saline at 37 ^∘^C. The potentiostat was a Gamry Reference 600+ with Framework$^\mathrm{TM}$ version 7.07. The frequency range was 40 mHz to 100 kHz. A 10 mV rms sinusoidal perturbation was applied at open circuit or at potentials ranging from −0.4 to +0.6 V(Ag|AgCl), and the resulting current response was measured. To facilitate analysis of the error structure, three replicate spectra were collected at each condition studied.

**Figure 1.**
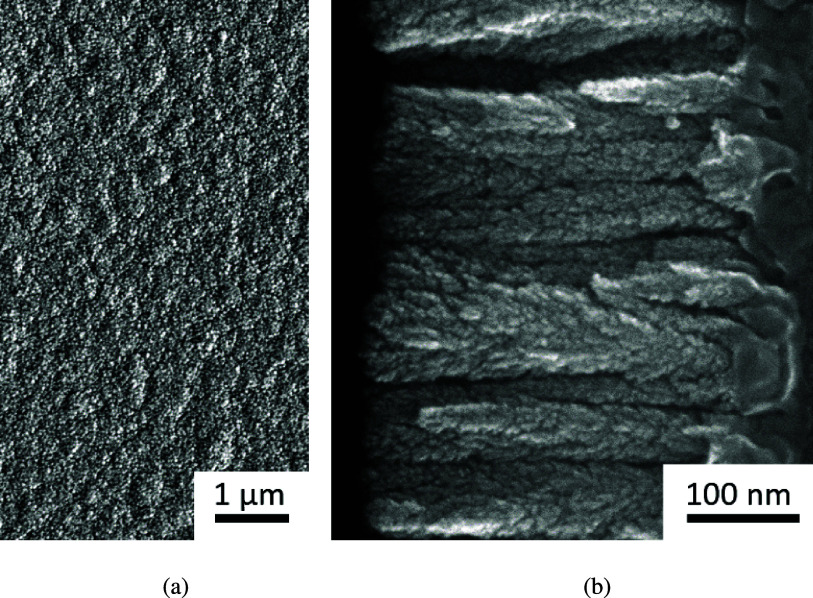
SEM images of the SIROF electrodes: (a) top view and (b) cross-sectional view.

Scanning electron micrographs of the SIROF deposition are shown in figure 1. The top view shown in figure 1(a) suggests that the surface has a roughened texture, and the side view shown in figure 1(b) reveals a columnar structure, consistent with a porous electrode.

The Gamry Reference 600+ potentiostat was used to perform CV on the same 2000 *µ*m^2^ SIROF micro-electrode used in the impedance analysis. The cyclic voltammograms were collected for potentials between −0.6 and +0.8 V(Ag|AgCl) at sweep rates of 50 mV s^−1^ and 50 V s^−1^ under the same conditions as used for the impedance measurements.

### 2.2. Data analysis

The SIROF EIS data were analyzed using Version 1.8 of the measurement model program published by Watson and Orazem [20] following the procedure outlined by Orazem [21]. The measurement model employed in the present work (see Agarwal *et al* [17, 22]) predates the linear Kramers–Kronig transform test presented by Boukamp [23] and has the advantage that it allows quantification of both stochastic and bias components of the error structure.

The real and imaginary impedance data as a function of frequency were uploaded to the measurement model program. The experimental noise level of the measurements were determined by fitting the generalized measurement model

\begin{align*} Z &amp; = R_\mathrm{e} + \sum_\mathit{k}^\mathit{K}\frac{R_\mathit{k}}{1 + \mathrm{j} \omega \tau_\mathit{k}} = R_\mathrm{e} + \frac{R_1}{1 + \mathrm{j} \omega \tau_1} + \frac{R_2}{1 + \mathrm{j} \omega \tau_2} \nonumber\\ &amp;\quad + \dots+ \frac{R_\mathit{K}}{1 + \mathrm{j} \omega \tau_\mathit{K}}\end{align*}

to each spectrum to obtain residuals as a function of frequency. The time constants $\tau_\mathit{k}$ can be expressed in terms of capacitance $C_\mathit{k}$ and resistance $R_\mathit{k}$ by

\begin{equation*} \tau_\mathit{k} = R_\mathit{k}C_\mathit{k}.\end{equation*}

The maximum number of Voigt circuit elements, $\mathit{K}$, used for regression was assessed by requiring that the 95.4% or $\pm 2\sigma$ confidence interval for each parameter may not include zero.

The measurement model served to filter lack of replication, and the standard deviation of the residuals for each set of replicates provided an estimate for the stochastic contribution to the measurement. These standard deviations were modeled by

\begin{equation*} \sigma_{\mathrm{r}} = \sigma_{\mathrm{j}} = \sigma = \alpha_\sigma |Z_\mathrm{j}| + \beta_\sigma |Z_\mathrm{r}| + \gamma_\sigma |Z|^2 + \delta_\sigma\end{equation*}

where *σ* is the standard deviation of errors, $|Z_\mathrm{j}|$ is the absolute value of the imaginary component of the impedance, $|Z_\mathrm{r}|$ is the absolute value of the real component of the impedance, and $|Z|$ is the magnitude of the impedance [21]. The parameters *α*_*σ*_, *β*_*σ*_, *γ*_*σ*_, and *δ*_*σ*_ are constants obtained by linear regression weighted by the estimated variance of the standard deviations. A unique error structure was identified for each set of three replicates at the various potentials studied.

Subsequent regressions were performed under error-structure weighting. Assessment of agreement with the Kramers–Kronig relations was conducted to determine the allowable frequency range of analysis in accordance with previous work [19, 21]. Origin^®^ 2023b software was used to calculate capacitances and generate the visual representations of the model regression results in this work.

### 2.3. Interpretation model

The model development includes the contribution of the $\mathrm{Ir}^{3+}/\mathrm{Ir}^{4+}$ redox couple, the impedance of a porous electrode, and the impedance associated with the surface of the porous electrode.

#### Contribution of the $\mathrm{Ir}^{3+}/\mathrm{Ir}^{4+}$ couple

2.3.1.

The oxidation/reduction reaction may be expressed as

\begin{align*} \mathrm{Ir}^{3+}\rightleftarrows\mathrm{Ir}^{4+}+\mathrm{e}^{-}.\end{align*}

Under the assumption that the oxidizable states are both finite in number and fixed in location, the total concentration of sites may be expressed as

\begin{equation*} c_\mathrm{tot} = c_3+c_4\end{equation*}

where *c*_3_ is the concentration of $\mathrm{Ir}^{3+}$ and *c*_4_ is the concentration of $\mathrm{Ir}^{4+}$. A corresponding surface concentration may be expressed as

\begin{equation*} \Gamma_\mathrm{tot} = \int_0^\delta c_\mathrm{tot} \mathrm{d}y.\end{equation*}

A similar integration may be performed for *c*_3_ and *c*_4_, yielding Γ_3_ and Γ_4_, respectively. The fractional occupation of a site by $\mathrm{Ir}^{3+}$ may be expressed as

\begin{equation*} \gamma = \frac{\Gamma_3}{\Gamma_\mathrm{tot}}\end{equation*}

and the fractional occupation of a site by $\mathrm{Ir}^{4+}$ may be expressed as

\begin{equation*} \frac{\Gamma_4}{\Gamma_\mathrm{tot}} = 1-\gamma.\end{equation*}

The oxidation current is given by

\begin{equation*} i_3 = K_3\gamma\exp\left(b_3V\right)\end{equation*}

where *K*_3_ is a rate constant with units of current density, the coefficient *b*_3_ is proportional to the apparent transfer coefficient, and $V = \Phi_\mathrm{m}-\Phi_0$ is the local potential driving force for the reaction. The equilibrium potential for the reaction is embedded in *K*_3_. The reduction current may be expressed as

\begin{equation*} i_4 = -K_4\left(1-\gamma\right)\exp\left(-b_4V\right)\end{equation*}

where *K*_4_ is a rate constant with units of current density, and the coefficient *b*_4_ is proportional to the apparent transfer coefficient. A conservation equation may be developed for the fractional occupation of sites by $\mathrm{Ir}^{3+}$ as

\begin{equation*} \mathrm{F}\Gamma_\mathrm{tot}\frac{\mathrm{d}\gamma}{\mathrm{d}t} = -\left(i_3+i_4\right)\end{equation*}

where $\mathit{F}$ is Faraday’s constant ($\mathit{F} = 96\,487$ C/equiv.). Under equilibrium conditions, $i_3+i_4 = 0$, and the fractional occupation of sites by $\mathrm{Ir}^{3+}$ may be expressed as

\begin{equation*} \overline{\gamma} = \frac{K_4\exp\left(-b_4\overline{V}\right)} {K_3\exp\left(b_3\overline{V}\right)+K_4\exp\left(-b_4\overline{V}\right)}.\end{equation*}

The equilibrium fractional occupation of sites by $\mathrm{Ir}^{3+}$ tends toward unity at negative potentials and toward zero at positive potentials. At $\overline{V} = 0$, $\gamma = K_4/(K_3+K_4)$, consistent with the observation that the equilibrium potential is included in the definitions of *K*_3_ and *K*_4_.

The impedance response associated with reaction (4) may be expressed as

\begin{equation*} Z_\mathrm{F} = \frac{\widetilde{V}}{\widetilde{i}_\mathrm{F}}\end{equation*}

where

\begin{equation*} V = \overline{V}+\mathrm{Re}\left\{\widetilde{V}\exp\left(\mathrm{j}\omega t\right)\right\}\end{equation*}

and $\widetilde{V}$ is the potential phasor. Similar expressions may be defined for *γ*, $i_\mathrm{F}$, *i*_3_, and *i*_4_. For small perturbation amplitudes, the current density phasors may be expressed as

\begin{equation*} \widetilde{i}_3 = b_3K_3\overline{\gamma}\exp\left(b_3\overline{V}\right)\widetilde{V} + K_3\exp\left(b_3\overline{V}\right)\widetilde{\gamma}\end{equation*}

and

\begin{equation*} \widetilde{i}_4 = b_4K_4\left(1-\overline{\gamma}\right)\exp\left(-b_4\overline{V}\right)\widetilde{V} + K_4\exp\left(-b_4\overline{V}\right)\widetilde{\gamma}\end{equation*}

The conservation equation (11) yields

\begin{equation*} \mathrm{j}\omega\mathit{F}\Gamma_\mathrm{tot}\widetilde{\gamma} = -\left(\widetilde{i}_3+\widetilde{i}_4\right)\end{equation*}

or

\begin{align*} \mathrm{j}\omega\mathit{F}\Gamma_\mathrm{tot}\widetilde{\gamma} &amp; = -\left(\frac{1}{R_\mathrm{t,3}}+\frac{1}{R_\mathrm{t,4}}\right)\widetilde{V} -\left(K_3\exp\left(b_3\overline{V}\right)\right. \nonumber\\ &amp;\left. \quad+ K_4\exp\left(-b_4\overline{V}\right)\right)\widetilde{\gamma}\end{align*}

where

\begin{equation*} R_\mathrm{t,3} = \frac{1}{b_3K_3\overline{\gamma}\exp\left(b_3\overline{V}\right)}\end{equation*}

and

\begin{equation*} R_\mathrm{t,4} = \frac{1}{b_4K_4\left(1-\overline{\gamma}\right)\exp\left(-b_4\overline{V}\right)}\end{equation*}

are charge-transfer resistances for the oxidation and reduction reactions, respectively.

As

\begin{equation*} i_\mathrm{F} = i_3+i_4\end{equation*}

combination of equations (15), (16), and (18) yields

\begin{align*} \frac{1}{Z_\mathrm{F}} &amp;= \left(\frac{1}{R_\mathrm{t,3}}+\frac{1}{R_\mathrm{t,4}}\right)\nonumber\\&amp;\quad \times \left[1-\frac{K_3\exp\left(b_3\overline{V}\right)+K_4\exp\left(-b_4\overline{V}\right)} {\mathrm{j}\omega\mathit{F}\Gamma_\mathrm{tot}+K_3\exp\left(b_3\overline{V}\right) +K_4\exp\left(-b_4\overline{V}\right)}\right].\end{align*}

The faradaic impedance may be expressed as

\begin{align*} {Z_\mathrm{F}} = R_\mathrm{t,eff}\left(\frac{\mathrm{j}\omega\mathit{F}\Gamma_\mathrm{tot}+{K_3\exp\left(b_3\overline{V}\right) +K_4\exp\left(-b_4\overline{V}\right)}}{\mathrm{j}\omega\mathrm{F}\Gamma_\mathrm{tot}}\right)\end{align*}

where $R_\mathrm{t,eff}$ is the effective resistance, given by

\begin{equation*} R_\mathrm{t,eff} = \frac{R_\mathrm{t,3}R_\mathrm{t,4}}{R_\mathrm{t,3}+R_\mathrm{t,4}}.\end{equation*}

Equation (23) may be rewritten as

\begin{equation*} Z_\mathrm{F} = R_\mathrm{t,eff}+\frac{1}{\mathrm{j}\omega C_\mathrm{t,eff}}\end{equation*}

where the effective capacitance, expressed as

\begin{equation*} C_\mathrm{t,eff} = \displaystyle\frac{\mathit{F}\Gamma_\mathrm{tot}}{R_\mathrm{t,eff} \left(K_3\exp\left(b_3\overline{V}\right)+K_4\exp\left(-b_4\overline{V}\right)\right)}\end{equation*}

is a function of potential, the concentration of oxidizable sites, and the associated rate constants.

**Figure 2.**
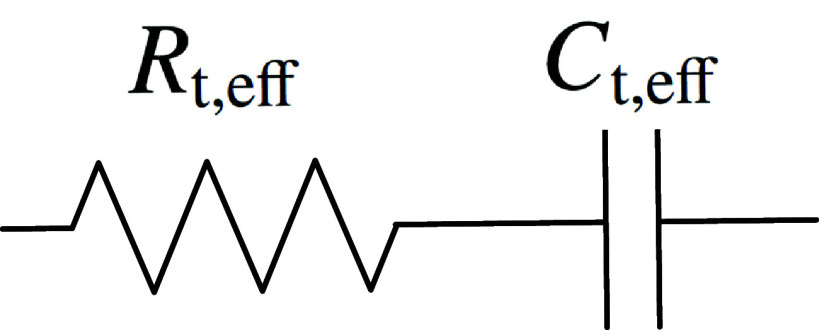
Electrical circuit representation for equation (25) as a series combination of an effective resistance and an effective capacitor.

The impedance given in equation (25) may be represented as an electrical circuit shown in figure 2. The electrical circuit element shown in figure 2 has been applied for dielectric relaxation in materials with a single time constant [24] where the element shown in figure 2 appears in parallel with the double-layer capacitance. A similar model was applied for deep-level states in semiconductors [25, 26]. The circuit developed in the present work differs from that used by Conway *et al* [15] who employ a parallel combination of a resistor and capacitor, and Sziráki and Bóbics [16], who assign a finite-length diffusion element to proton hopping between the adjacent oxide ions on the lattice.

**Figure 3.**
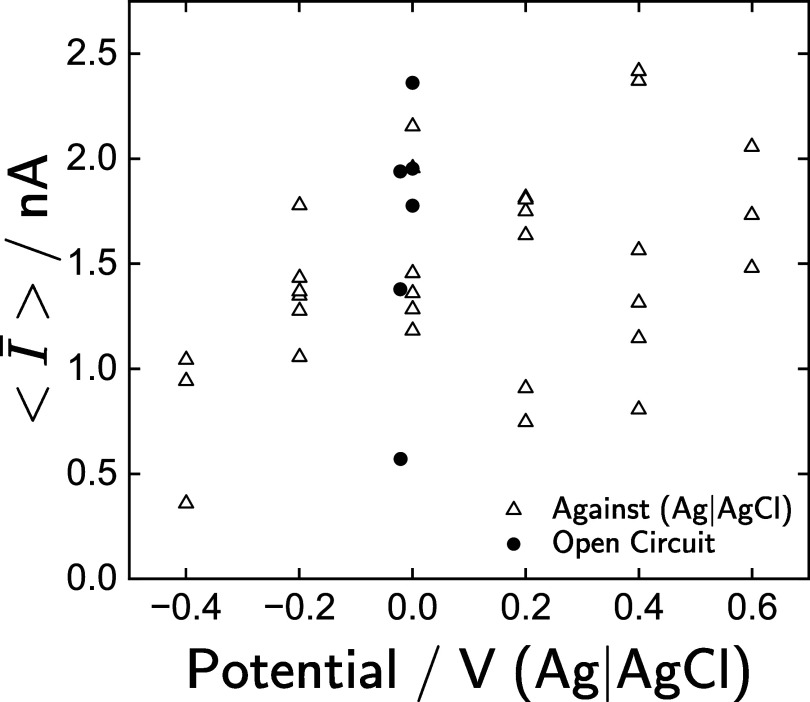
Average steady-state current values as a function of potential for all 36 collected EIS spectra. Numerical values are provided in table 1.

The average steady-state current values $\langle\bar{I}\rangle$, shown in figure 3 as a function of potential, cannot be differentiated from the current measured at open circuit, where the total current is expected to be zero. The data presented in figure 3 suggest the absence of a steady-state faradaic current, as would be expected for experiments performed for potentials within the water window. Equation (25) shows that the contribution of the Ir^3+^/Ir^4+^ reactions will tend toward zero at steady state. Thus, the impedance response can be attributed to a combination of double-layer charging current and the faradaic reaction associated with the Ir^3+^/Ir^4+^ transitions.

The physical properties of IrO_2_ may be used to estimate the maximum charge density and capacitance that can be attributed to the $\mathrm{Ir}^{3+}/\mathrm{Ir}^{4+}$ couple. The data required for this calculation were obtained from the PubChem Compound Summary for CID 82 821, Iridium Dioxide [27]. The area-basis charge density of IrO_2_ is given by

\begin{equation*} q = 1000\frac{n\mathit{F}\rho\delta\left(1-\phi\right)}{M} = 107\;\mathrm{\textrm{mC cm}^{-2}}\end{equation*}

where *n* = 1 is the number of electrons transferred, $\mathit{F} = 96\,487$ Coul/equiv. is Faraday’s constant, *ρ* = 11.66 g cm^−3^ is the density, *M* = 224.22 g mol^−1^ is the molecular weight, *δ* = 320 nm is the film thickness, and the void fraction *φ* was assumed to have a value of $1/3$.

#### 2.3.2. Porous electrode

The SEM image shown in figure 1(b) suggests that a porous electrode model may be appropriate. Jurczakowski *et al* [28] showed that the impedance associated with gold porous electrodes could be described as the parallel contribution of surface and pore impedances. A similar approach was taken in the present work, i.e.

\begin{equation*} Z = R_\mathrm{e} + \frac{Z_\mathrm{flat}Z_\mathrm{pore}}{Z_\mathrm{flat}+Z_\mathrm{pore}}.\end{equation*}

**Figure 4.**
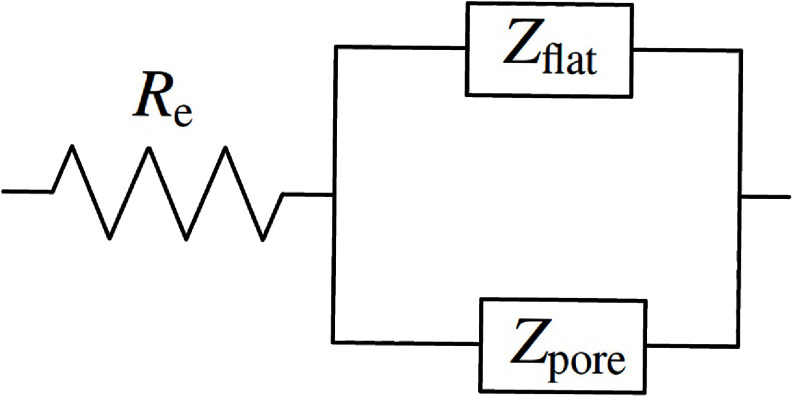
Circuit diagram representing the model equation (28) accounting for the contributions of the pore walls and the electrode surface.

The model accounting for the contributions of the pore walls and the electrode surface is presented as an electrical circuit in figure 4.

Following Lasia [29], the impedance for an electrode with cylindrical pores [30] was expressed as

\begin{equation*} Z_\mathrm{pore} = \frac{R_\mathrm{p}}{\sqrt{\Lambda}}\coth\left(\sqrt{\Lambda}\right)\end{equation*}

where $Z_\mathrm{pore} $ is the impedance of the porous electrode, $R_\mathrm{p}$ is a lumped parameter representing the resistance of a pore filled with electrolyte, i.e.

\begin{equation*} R_\mathrm{p} = \frac{A\rho_\mathrm{s}\ell}{\pi r^2 n}\end{equation*}

where *A* is the nominal area of the electrode, $\rho_\mathrm{s}$ is the resistivity of the solution in the pore, $\ell$ is the depth of the pore, *r* is the radius of the pore, and *n* is the number of pores. The parameter Λ is the dimensionless admittance

\begin{equation*} \Lambda = \frac{B}{Z_\mathrm{eq}}\end{equation*}

where *B* is a lumped parameter

\begin{equation*} B = \frac{2\rho_\mathrm{s}\ell^2}{r}\end{equation*}

and $Z_\mathrm{eq}$ is the impedance on the pore wall. In this case, the impedance on the pore wall is considered to be the parallel contribution of the impedance given by equation (25) and a constant-phase element. Thus,

\begin{equation*} Z_\mathrm{eq} = \frac{Z_\mathrm{cpe,p}Z_\mathrm{F}}{Z_\mathrm{cpe,p}+Z_\mathrm{F}}\end{equation*}

where

\begin{equation*} Z_\mathrm{cpe,p} = \frac{1}{\left(\mathrm{j}\omega\right)^{\alpha_\mathrm{p}}Q_\mathrm{p}}\end{equation*}

and parameters $\alpha_\mathrm{p}$ and $Q_\mathrm{p}$ are associated with the pore wall.

The impedance of the electrode surface was given as

\begin{equation*} Z_\mathrm{flat} = \frac{Z_\mathrm{cpe,f}Z_\mathrm{F}}{Z_\mathrm{cpe,f}+Z_\mathrm{F}}\end{equation*}

where

\begin{equation*} Z_\mathrm{cpe,f} = \frac{1}{\left(\mathrm{j}\omega\right)^{\alpha_\mathrm{f}}Q_\mathrm{f}}\end{equation*}

and parameters $\alpha_\mathrm{f}$ and $Q_\mathrm{f}$ are associated with the surface of the electrode. The influence of iridium oxidation and reduction was expressed as equation (25) with the same parameters used for the pore walls.

The present model comprises nine independent parameters, $R_\mathrm{e}$, $R_\mathrm{t,eff}$, $C_\mathrm{t,eff}$, $R_\mathrm{p}$, *B*, $\alpha_\mathrm{p}$, $\alpha_\mathrm{f}$, $Q_\mathrm{p}$, and $Q_\mathrm{f}$. For some regressions, the number of parameters were reduced to ensure that all regressed parameters were statistically significant. For example, for the regression shown in table 3, the exponent $\alpha_\mathrm{p}$ was set to unity, consistent with a capacitive behavior, and a single value of *Q* was obtained, i.e. $Q_\mathrm{p} = Q_\mathrm{f}$.

## 3. Results

Interpretation of impedance spectroscopy data requires both a description of the chemistry and physics that govern the system and an assessment of the error structure of the measurement. The interpretation model was designed to account for the geometry and observed electrochemical properties of the electrode. Both error analysis and process model regression were performed using Version 1.8 of the measurement model program [20] using the step-by-step procedures described elsewhere [21].

### 3.1. Experimental results

The data used in this analysis are summarized in table 1. The total time required for the measurements was 7.6 h, and the average time per impedance scan was 11 min. Potential was allowed to stabilize for a period of one minute before each set of replicates was measured. Potential and current values reported are the numerical average of values recorded at each frequency. Potential is reported in units of V(Ag|AgCl) and the current is reported in units of nA. Measurements N and Y were performed at open circuit. The change in measured potential from −0.0209 V(Ag|AgCl) for N to +0.0004 V(Ag|AgCl) for Y suggests nonstationary behavior, consistent with the need to remove some low-frequency data. The insensitivity of measured current to potential suggests that the steady current was effectively equal to zero.

**Table 1.** SIROF data used in the present analysis in temporal sequence. The total time required for the measurements was 7.6 h, and the average time per impedance scan was 11 min. The value of time reported is the elapsed time following initiation of the exerimental sequence. Potential and current values reported are the numerical average of values recorded at each frequency. Potential is referenced to the Ag|AgCl electrode. Measurements N and Y were performed at open circuit. The change in measured potential from −0.0209 V(Ag|AgCl) for N to +0.0004 V(Ag|AgCl) for Y suggests nonstationary behavior, consistent with the need to remove some low frequency data.

	Replicate:	1		2		3	
Identifier	Time, min	Potential, V	Current, nA	Potential, V	Current, nA	Potential, V	Current, nA
N (OC)	0	−0.02 094	0.57	−0.02 096	1.38	−0.02 096	1.94
O	40	0.19 999	0.91	0.19 999	1.64	0.19 999	1.75
P	78	0.39 982	0.81	0.39 981	2.37	0.39 981	1.56
Q	117	0.59 967	1.48	0.59 967	2.06	0.59 967	1.73
R	157	0.39 981	1.15	0.39 981	1.31	0.39 982	2.42
S	196	0.19 999	0.75	0.20 000	1.81	0.19 999	1.81
T	233	0.00 015	1.36	0.00 015	2.16	0.00 015	1.18
U	273	−0.19 956	1.28	−0.19 956	1.43	−0.19 956	1.06
V	310	−0.39 939	0.36	−0.39 940	1.043	−0.39 940	0.94
W	352	−0.19 956	1.35	−0.19 956	1.37	−0.19 956	1.78
X	388	0.00 014	1.46	0.00 014	1.96	0.00 015	1.28
Y (OC)	429	0.00 040	1.95	0.00 040	2.36	0.00 040	1.78

**Figure 5.**
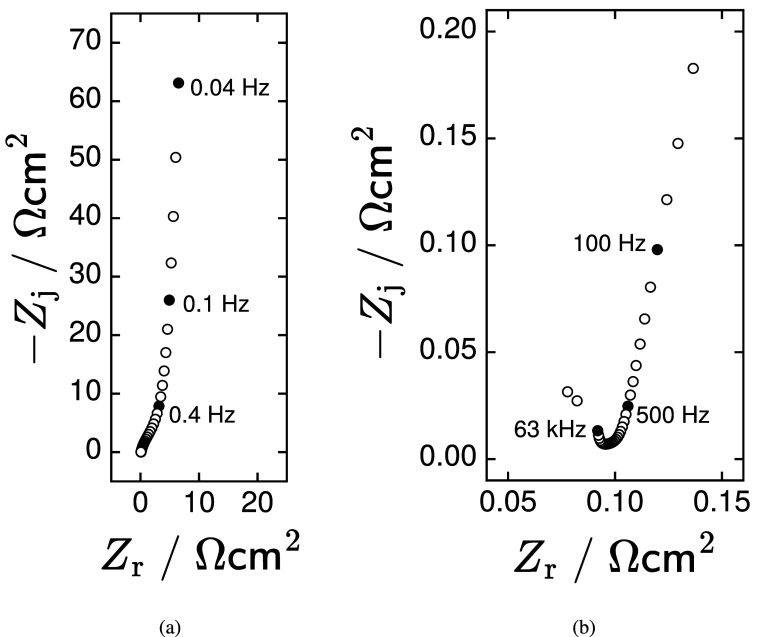
Impedance results for data set N1 in Nyquist format: (a) complete spectrum and (b) zoom into the high-frequency region. The frequency range was 40 mHz–100 kHz, and the measurement was performed at open circuit.

A typical impedance scan, shown in Nyquist format in figure 5(a), is consistent with porous electrode behavior. These data were collected at open circuit. The high-frequency region of the data, presented in figure 5(b), shows that the two highest frequencies measured are outliers. These needed to be removed before successful regression was possible. The subsequent upturn in the impedance can be attributed to the contribution of nonuniform current and potential. The resulting ohmic impedance is addressed in section 3.2.3.

### 3.2. Analysis of error structure

The assessment of error structure included determination of the stochastic error structure, used to weight subsequent regressions, and assessment of lack of consistency with the Kramers–Kronig relations, generally caused by nonstationary behavior. In addition, data were found to be influenced by the frequency-dependent ohmic impedance associated with geometry-induced nonuniform current and potential distributions. This preliminary assessment made use of the measurement model given by equation (1).

#### 3.2.1. Assessment of stochastic errors

The measurement model (equation (1)) was fit to each measurement within a set of replicates N through Y in table 1 using modulus weighting. The evaluation of the corresponding standard deviation required that the number of Voigt elements was equal for each of the replicates and that the frequency range was identical. The standard deviation of the residuals was calculated as a function of frequency for each set of replicates.

**Figure 6.**
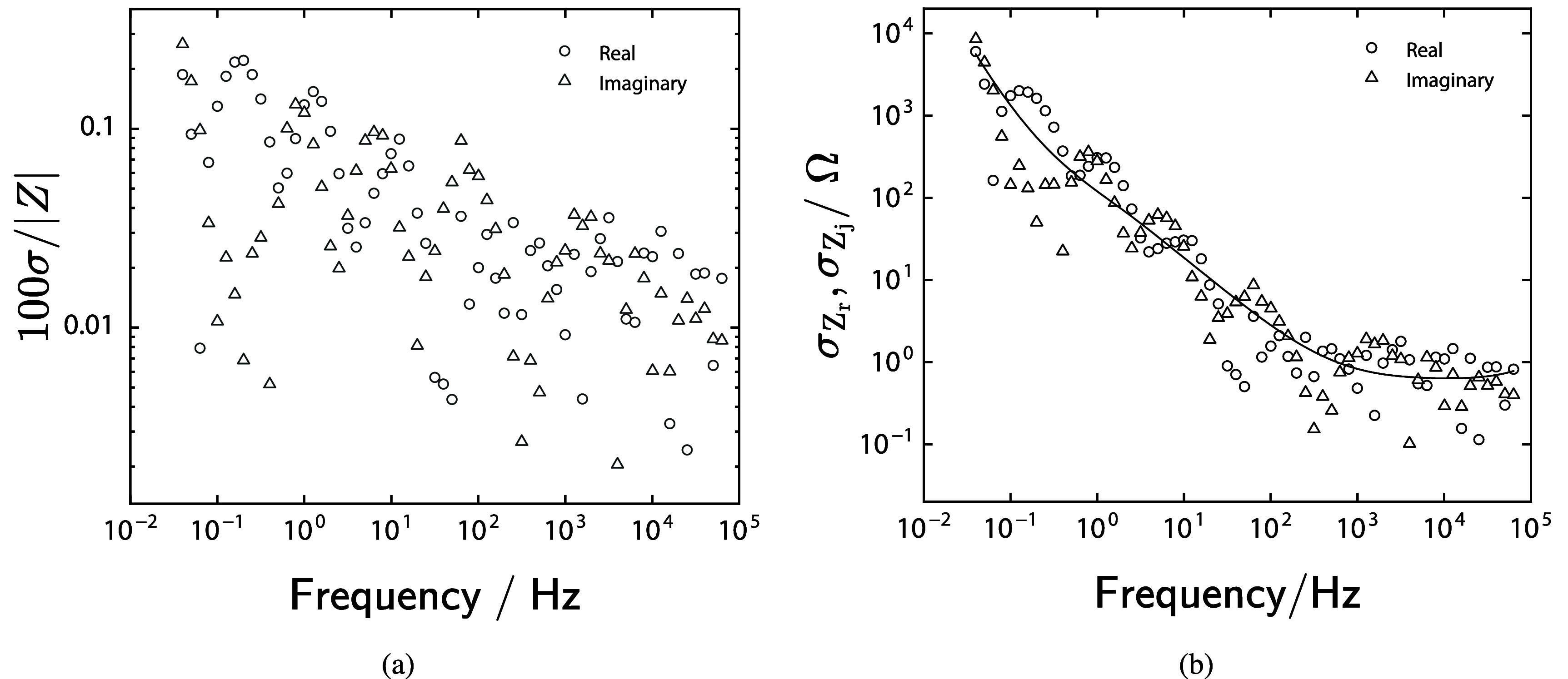
Error analysis plots for data set N, measured at open circuit (see table 1) as functions of frequency: (a) standard deviations normalized by the averaged modulus and multiplied by 100 to yield a percentage and (b) non-normalized standard deviations. The line represents the fit of equation (3), $\bigcirc$ represents the standard deviation for the real part of the impedance and $\triangle$ represents the standard deviation for the imaginary part of the impedance.

The resulting standard deviations are presented for Case N (open circuit) in figure 6 as a function of frequency. The standard deviation normalized by the modulus is presented as a percentage in figure 6(a). The normalized standard deviation is highest at low frequency, but is always below 0.3%, consistent with previous measurements with good signal to noise [31]. The non-normalized standard deviations are presented in figure 6(b). The standard deviations of the real and imaginary components overlap as is expected for data sets that satisfy the Kramers–Kronig relations.

The line in figure 6(b) represents the fit of equation (3). The parameters used to obtain the fit result for the three spectra in set N are shown in table 2. A statistically significant value could not be obtained for *β*_*σ*_. A total of 12 unique error structures were developed, one for each set of replicate spectra.

**Table 2.** Error structure parameters for data set N (see equation (3)).

Parameter	Value ± St. Dev.
*α*_*σ*_	$4.65\times10^{-4}$ ± $7.5\times10^{-20}$
*β*_*σ*_	−
*γ*_*σ*_	$3.98\times10^{-10}$ ± $1.3\times10^{-13}$
*δ*_*σ*_	0.462 ± 0.071

#### 3.2.2. Assessment of consistency with the Kramers–Kronig relations

The two highest frequency data were found consistently to be outliers and were removed for all regressions. The generalized measurement model, equation (1), was fit to data using the inverse of the measured variance (see figure 6 and table 2) to weight regressions. As the measurement model is consistent with the Kramers–Kronig relations, the ability to fit impedance data by the measurement model provides an indication that the data are consistent with the Kramers–Kronig relations. While Agarwal *et al* [19] have recommended that the assessment of consistency with the Kramers–Kronig relations is best performed by fitting to the real and imaginary parts of the impedance separately, that advice is best suited for data that approach the zero-frequency asymptote. For the present data, consistency with the Kramers–Kronig relations was assessed by performing a complex fit of equation (1) to the data.

**Figure 7.**
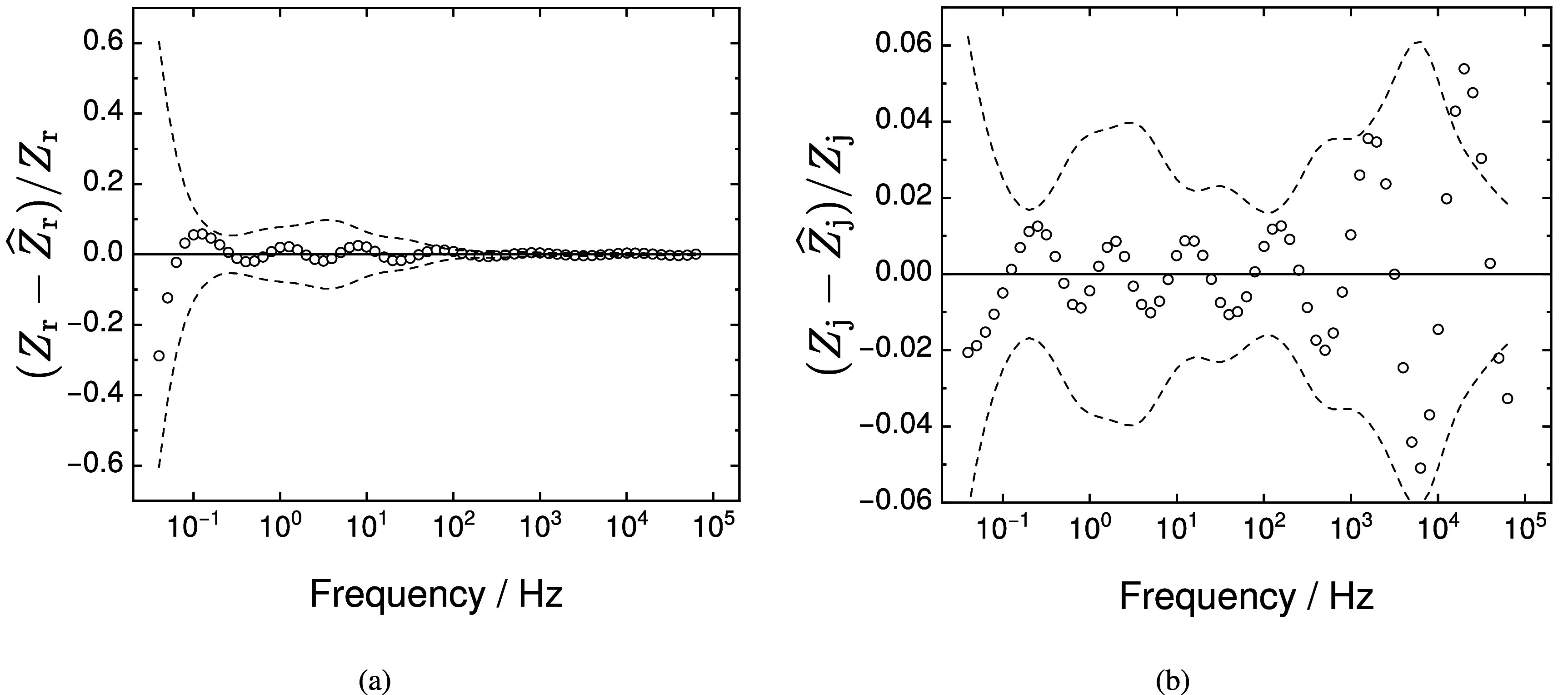
Normalized residual error for complex regression of the measurement model with seven Voigt elements to the impedance for data N1: (a) real residual errors and (b) imaginary residual errors. The regression was weighted in accordance to the stochastic error structure identified for data set N1–N3. Dashed lines represent the bounds of the 95.4% confidence interval for the model. Data that fall outside the confidence interval may be considered to be inconsistent with the Kramers–Kronig relations.

The measurement model was fit to the real and imaginary parts of the impedance. A Monte Carlo simulation was performed to estimate the $\pm2\sigma$ (95.4%) confidence interval for the model value. As shown in figure 7, normalized errors were inside the confidence interval at low frequency. The maximum number of Voigt elements was determined by requiring that the 95.4% or $\pm 2\sigma$ confidence interval for each parameter may not include zero. In this case, seven Voigt elements could be resolved.

A similar analysis of the other 35 spectra identified showed that the low-frequency data were consistent with the Kramers–Kronig relations, with the exception of data set Y (see table 1), which required removal of the lowest frequency measured. As shown in table 1, the measured open-circuit potential changed over a period of 7.6 h from −0.0209 to +0.0004 V(Ag|AgCl), but this shift was not sufficient to cause the measured impedance to be inconsistent with the Kramers–Kronig relations.

#### 3.2.3. Assessment of ohmic impedance

The geometry-induced nonuniform current and potential distributions give rise to a frequency-dependent contribution to the impedance that is associated with ohmic characteristics of the electrode–electrolyte interface. This contribution, termed the ohmic impedance [32], appears at frequencies above

\begin{equation*} f_\mathrm{c} = \frac{1}{2\pi R_\mathrm{e} C}\end{equation*}

where $R_\mathrm{e}$ is the high-frequency limit of the ohmic impedance. Regression of the measurement model yields estimates for the ohmic resistance $R_\mathrm{e}$ and capacitance, given as [33]

\begin{equation*} \frac{1}{C_\mathrm{MM}} = \sum_{k = 1}^{n}\frac{1}{C_\mathrm{k}}.\end{equation*}

Evaluation of the characteristic frequency given by equation (37) is iterative because the capacitance calculated by the measurement model includes the contribution of the ohmic impedance, i.e.

\begin{equation*} \frac{1}{C} = \frac{1}{C_\mathrm{SIROF}} + \frac{1}{C_\mathrm{ohmic}}\end{equation*}

resulting in a lower overall capacitance and a larger value for the characteristic frequency.

**Figure 8.**
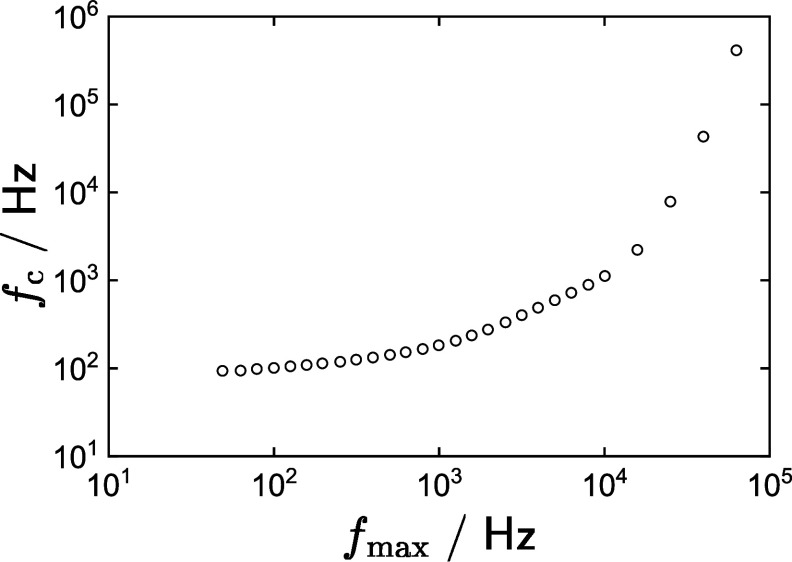
Characteristic frequency from equation (37) as a function of the maximum frequency included in the analysis. This iterative analysis provides the frequency above which ohmic impedance has a significant impact on the impedance spectra.

The characteristic frequency obtained from equation (37) is presented in figure 8 as a function of the maximum frequency, $f_\mathrm{max}$, included in the analysis. The influence of ohmic impedance is reduced when $f_\mathrm{max}$ is smaller than $f_\mathrm{c}$. In figure 8, $f_\mathrm{c}$ approaches $f_\mathrm{max}$ for frequencies between 90 and 100 Hz. While it is possible to include ohmic impedance in the process model, this approach requires an additional four parameters. The approach taken in the present work is to remove the high-frequency data that were affected by ohmic impedance, leaving a data set that is influenced solely by the electrochemistry of the electrode–electrolyte interface.

### 3.3. Interpretation model regression

**Figure 9.**
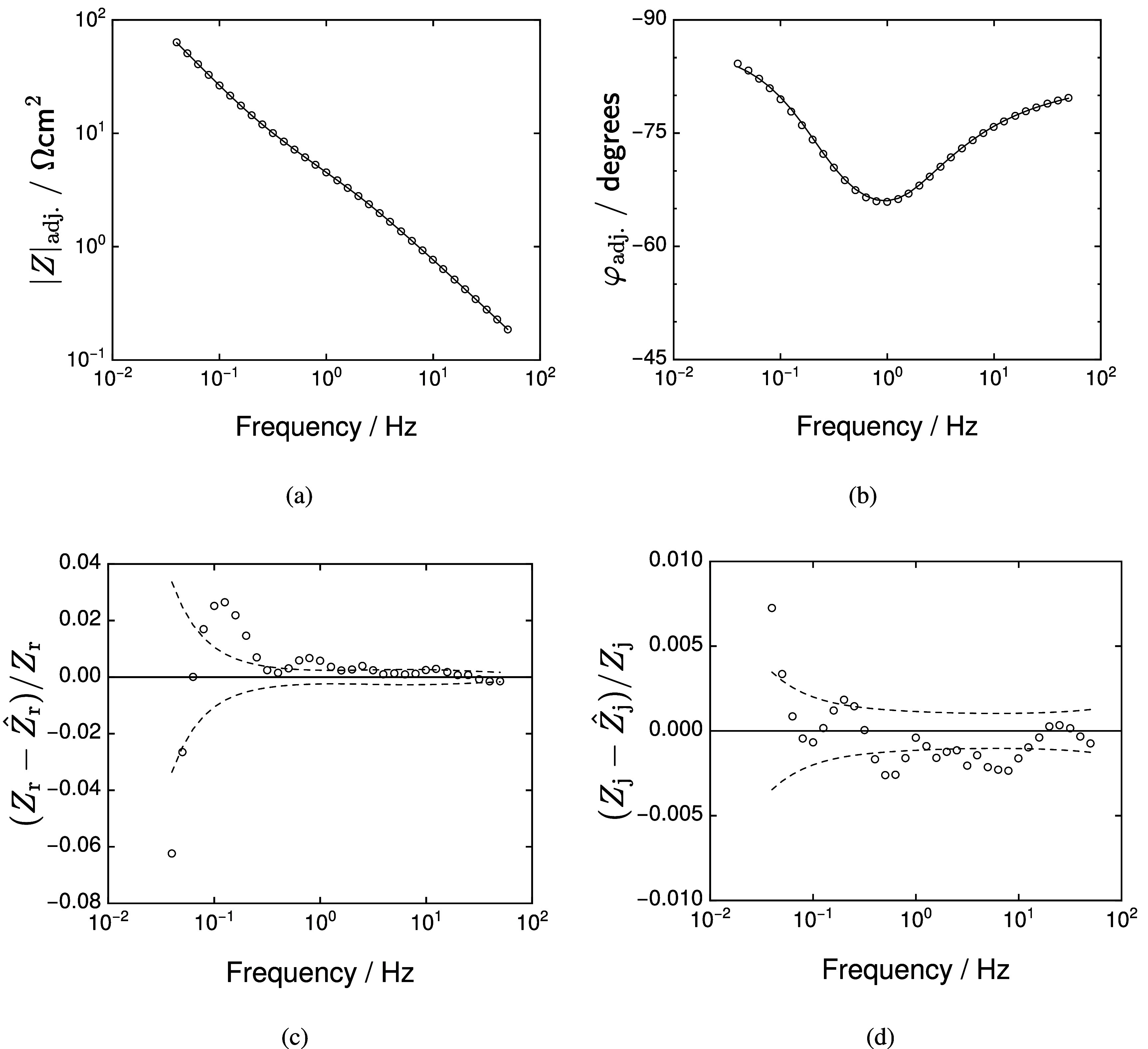
Regression results for the fit of equation (28) to data set N1 (see table 1): (a) and (b) ohmic-resistance-corrected magnitude and phase angle, respectively, as functions of frequency where the line represents the model fit and (c) and (d) real and imaginary normalized residuals, respectively, as functions of frequency where the upper and lower dashed lines represent the $\pm2\sigma$ for the stochastic error structure of the data represented by equation (3).

The model represented by equation (28) was fit to the impedance spectra for frequencies that were both below the ohmic impedance limit and in agreement with the Kramers–Kronig relations. The Levenberg–Marquardt regression was weighted by the error structure obtained for each set of replicates. The results of the fitting for data N1 are shown in figure 9. The ohmic-resistance-corrected magnitude

\begin{equation*} |Z|_\mathrm{adj} = \sqrt{\left(Z_\mathrm{r}-R_\mathrm{e}\right)^2 + Z_\mathrm{j}^2}\end{equation*}

and phase angle

\begin{equation*} \varphi_\mathrm{adj} = \tan^{-1}\left(\frac{Z_\mathrm{j}}{Z_\mathrm{r}-R_\mathrm{e}}\right)\end{equation*}

shown in figures 9(a) and (b), respectively, show that the model provides a good fit to the data. The real and imaginary normalized residuals, shown in figures 9(c) and (d), respectively, show that the normalized residuals fall within ± 1% for all frequencies. While not shown here, the model also shows a good fit to the data presented in Nyquist format. Figure 9 is representative of the fit quality for all 36 spectra.

The model equation (28) comprises six parameters, and each regression yielded statistically significant values for the six parameters. Parameter values obtained by the regression to data set N are provided in table 3. Values for the weighted $\chi^2/ \nu$ statistic, where *ν* is the degree of freedom for the regression, are also provided. A good fit is represented by

\begin{equation*} \chi^2/ \nu = 1+\sqrt{2/\nu}\end{equation*}

where, for the present work, *ν* is on the order of 48. Thus, a good fit would be represented by $\chi^2/\nu = 1.2$, suggesting that some improvement in the model may be possible. For most of the regressions performed in the present work, the $\chi^2/ \nu$ values varied from 1 to 600. These $\chi^2/ \nu$ values are in the range of previously published models for other electrochemical systems, e.g. 53 to 139 for Wang *et al* [34] and 7 to 13 for You *et al* [35]. The model encompassing a parallel combination of the flat and porous components of impedance provided a good fit to the SIROF micro-electrode EIS data.

**Table 3.** Extracted model parameters and $\chi^2/ \nu$ obtained by regression of equation (28) to replicates for data set N (see table 1). The confidence interval reported is ±*σ*. For these regressions, $Q_\mathrm{p} = Q_\mathrm{f}$ and $\alpha_\mathrm{p}$ was fixed to unity.

Parameter data set	N1	N2	N3
$R_\mathrm{e}$ / $\Omega\mathrm{cm}^2$	0.10 323 ± 0.00 042	0.10 310 ± 0.00 030	0.10 312 ± 0.00 031
$R_\mathrm{p}$ / $\Omega\mathrm{cm}^2$	44.15 ± 0.95	45.10 ± 0.68	45.37 ± 0.71
*B* / $\Omega\mathrm{cm}^2$	33.18 ± 0.39	34.10 ± 0.28	34.47 ± 0.30
$Q_\mathrm{f}$ / $\mathrm{mF}\mathrm{cm}^{-2}\mathrm{s}^{\alpha-1}$	26.01 ± 0.18	25.21 ± 0.12	24.84 ± 0.12
$Q_\mathrm{p}$ / $\mathrm{mF}\mathrm{cm}^{-2}\mathrm{s}^{\alpha-1}$	−	−	−
$\alpha_\mathrm{p}$	1	1	1
$\alpha_\mathrm{f}$	0.9174 ± 0.0011	0.91 851 ± 0.00 081	0.91 897 ± 0.00 084
$R_\mathrm{t,eff}$ / $\Omega\mathrm{cm}^2$	20.24 ± 0.45	20.41 ± 0.31	20.77 ± 0.33
$C_\mathrm{t,eff}$, $\mathrm{mF}\,\mathrm{cm}^{-2}$	8.53 ± 0.26	8.60 ± 0.18	8.56 ± 0.19
$\chi^2/ \nu$	28	13	14

Calculated			

$C_\mathrm{flat}$, $\mathrm{mF}\,\mathrm{cm}^{-2}$	15.26 ± 0.24	14.87 ± 0.16	14.68 ± 0.17
$C_\mathrm{pore}$, $\mathrm{mF}\,\mathrm{cm}^{-2}$	26.01 ± 0.18	25.21 ± 0.12	24.84 ± 0.12
$C_\mathrm{total}$, $\mathrm{mF}\,\mathrm{cm}^{-2}$	58.33 ± 0.94	57.28 ± 0.65	56.64 ± 0.67

## 4. Discussion

The potential dependence of regressed parameters shows a coherent trend. In particular, the pore-wall and surface capacitances obtained from impedance show a striking potential dependence. These values are compared to the values of capacitance extracted from CV data. The value obtained at large sweep rates is in agreement with those obtained from impedance data once the potential range is modified to account for the influence of ohmic potential drop.

### 4.1. Potential dependence of regressed parameters

**Figure 10.**
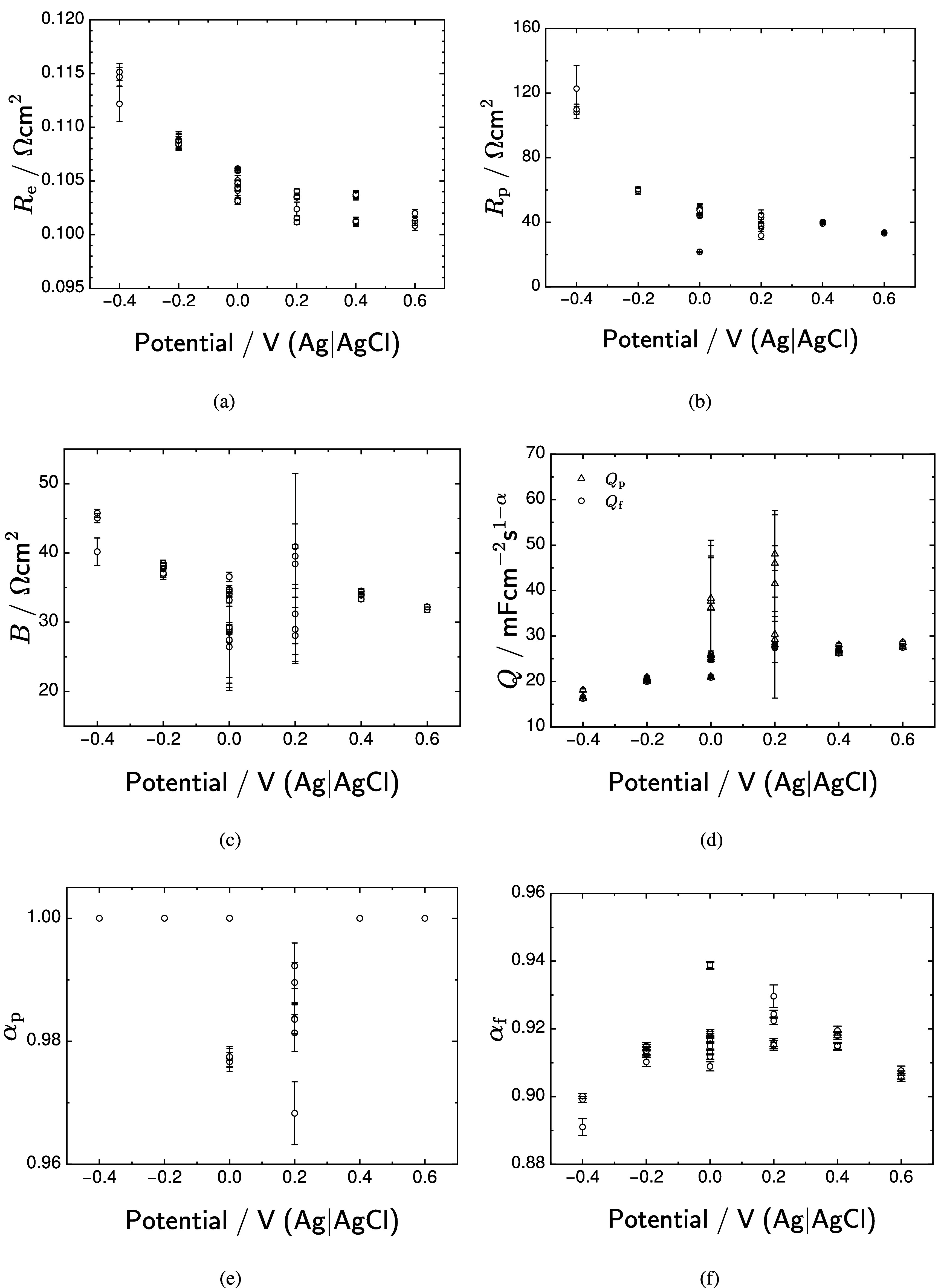
Regressed values for model parameters as functions of potential: (a) ohmic resistance $R_\mathrm{e}$; (b) lumped pore resistance $R_\mathrm{p}$; (c) lumped pore geometry parameter *B*; (d) CPE coefficient *Q*; (e) pore CPE exponent $\alpha_\mathrm{p}$; and (f) surface CPE exponent $\alpha_\mathrm{f}$. Error bars represent one standard deviation.

The consistency of the extracted model parameters and their dependence on potential was assessed by plotting the model parameters from each regression as a function of potential. The scatter plots of the six model parameters are presented in figure 10 as functions of potential. The extracted model parameter values for a given potential are consistent for EIS measurements separated in the temporal sequence. The best examples of this consistency are the parameter values at 0 V(Ag|AgCl) since there were 4 measurements spread out over 7 h and the parameter values remained consistent. All error bars represent one standard deviation. The ohmic resistance given in figure 10(a) decreased smoothly with increasing potential from 0.115 $\Omega\mathrm{cm}^2$ at a potential of −0.4 V(Ag|AgCl) to 0.102 $\Omega\mathrm{cm}^2$ at a potential of 0.6 V(Ag|AgCl). The pore resistance, given in equation (30), also decreased with potential, as shown in figure 10(b). The pore resistance varied from 41 $\Omega\mathrm{cm}^2$ at a potential of −0.4 V(Ag|AgCl) to 15 $\Omega\mathrm{cm}^2$ at a potential of 0.6 V(Ag|AgCl). The pore geometry parameter *B*, given by equation (32) and presented in figure 10(c), decreases with increasing potential.

The constant-phase element coefficient, *Q*, presented in figure 10(d), increased with increasing potential. Independent values could be obtained for $Q_\mathrm{p}$ and $Q_\mathrm{f}$ only at potentials of 0 and 0.2 V(Ag|AgCl). The constant-phase-element exponent for the pore walls, $\alpha_\mathrm{p}$, is presented in figure 10(e) as a function of potential. Values different from unity were found only at potentials of 0 and 0.2 V(Ag|AgCl). In contrast, the constant-phase-element exponent for the electrode surface, $\alpha_\mathrm{f}$, shows clear dependence on potential, as shown in figure 10(f). The values of $\alpha_\mathrm{f}$ increased with increasing potential from −0.4 to 0.2 V(Ag|AgCl) up to a maximum of approximately 0.92. The value of $\alpha_\mathrm{f}$ then decreased with increasing potential to an approximate value of 0.91 at 0.6 V(Ag|AgCl).

**Figure 11.**
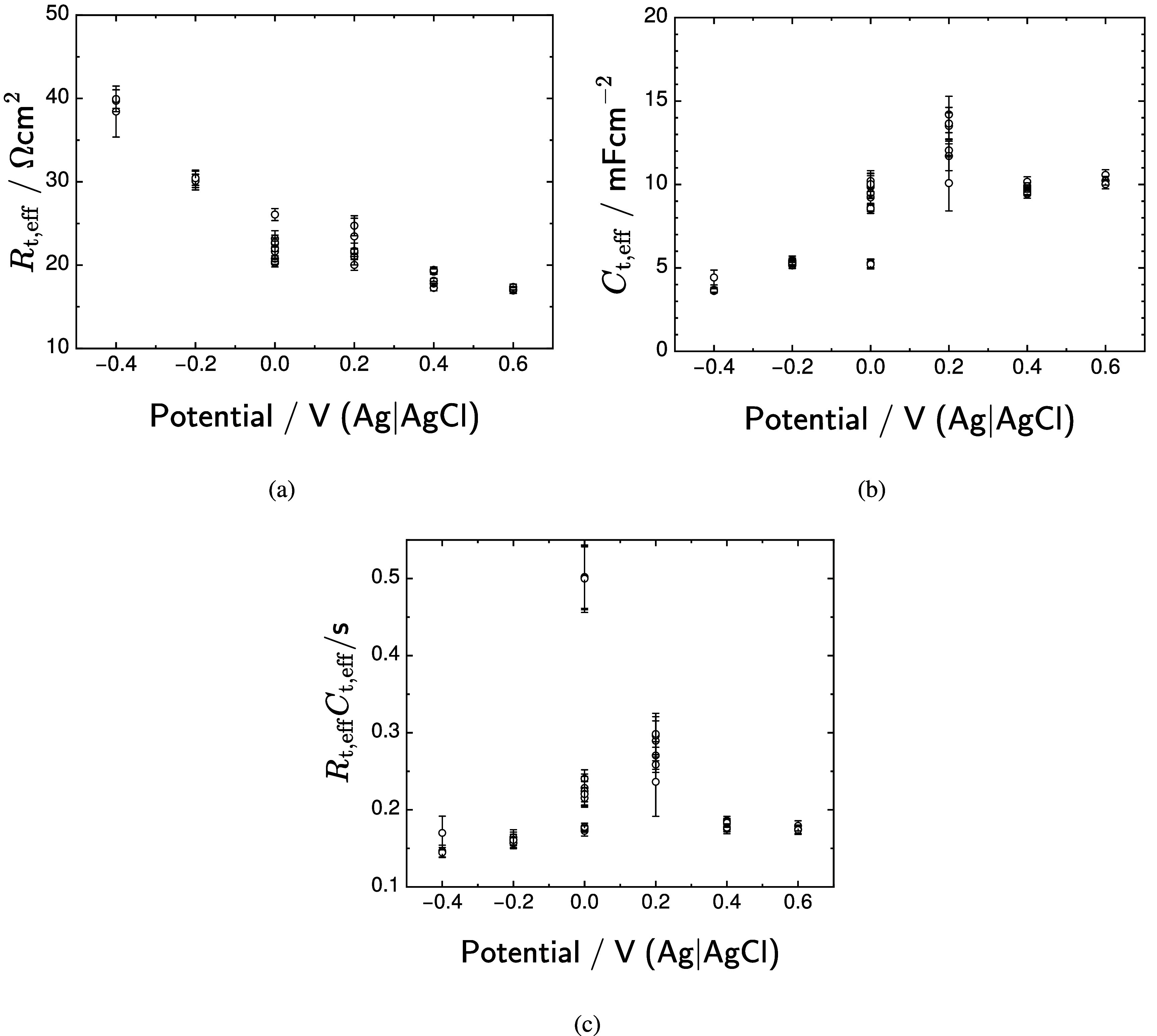
Regressed values for model parameters associated with changes in the iridium oxidation state as functions of potential: (a) resistance $R_\mathrm{t,eff}$; (b) capacitance $C_\mathrm{t,eff}$; and (c) time constant $R_\mathrm{t,eff}C_\mathrm{t,eff}$. Error bars represent one standard deviation.

The parameters associated with changes in oxidation state for iridium are presented in figure 11. The value of $R_\mathrm{t,eff}$, given as equation (24), decreased with increasing potential, as shown in figure 11(a). The value of $C_\mathrm{t,eff}$, given as equation (26), increased with increasing potential, as shown in figure 11(b). The $R_\mathrm{t,eff}C_\mathrm{t,eff}$ time constant had a value of 0.15 s, increasing slightly at potentials of 0 and 0.2 V(Ag|AgCl). The time constant is sufficiently small that a rest period of 1 min between measurements would be sufficient to allow equilibration at each applied potential.

### 4.2. Assessment of capacitance from impedance

Two methods were used to estimate the electrode capacitance from the impedance results. The capacitance may be obtained from the measurement model using equation (38). The second approach, conversion of the CPE parameters to a capacitance, requires an assumption of a type of time-constant distribution [36–38]. Assumption of a normal distribution of time constants through a film [33, 39] yielded non-physical values for film thickness on the order of 0.002 nm. Therefore, the CPE behavior was attributed to a surface distribution of time constants. The effective double-layer capacitance for the electrode surface was obtained from the formula derived by Brug *et al* [40], i.e.

\begin{equation*} C_\mathrm{f,eff,dl} = Q_\mathrm{f}^{1/\alpha_\mathrm{f}}R_\mathrm{e}^{\left(1-\alpha_{\mathrm{f}}\right)/\alpha_{\mathrm{f}}}\end{equation*}

where $R_\mathrm{e}$ is the ohmic resistance. An analogous formula was applied for the pore walls employing the pore resistance $R_\mathrm{p}$ instead of the ohmic resistance, e.g.

\begin{equation*} C_\mathrm{p,eff,dl} = Q_\mathrm{p}^{1/\alpha_\mathrm{p}}R_\mathrm{p}^{\left(1-\alpha_{\mathrm{p}}\right)/\alpha_{\mathrm{p}}}.\end{equation*}

The total capacitances for the pore wall and the flat surface were obtained by adding the capacitance $C_\mathrm{t,eff}$ associated with change of iridium oxidation states.

**Figure 12.**
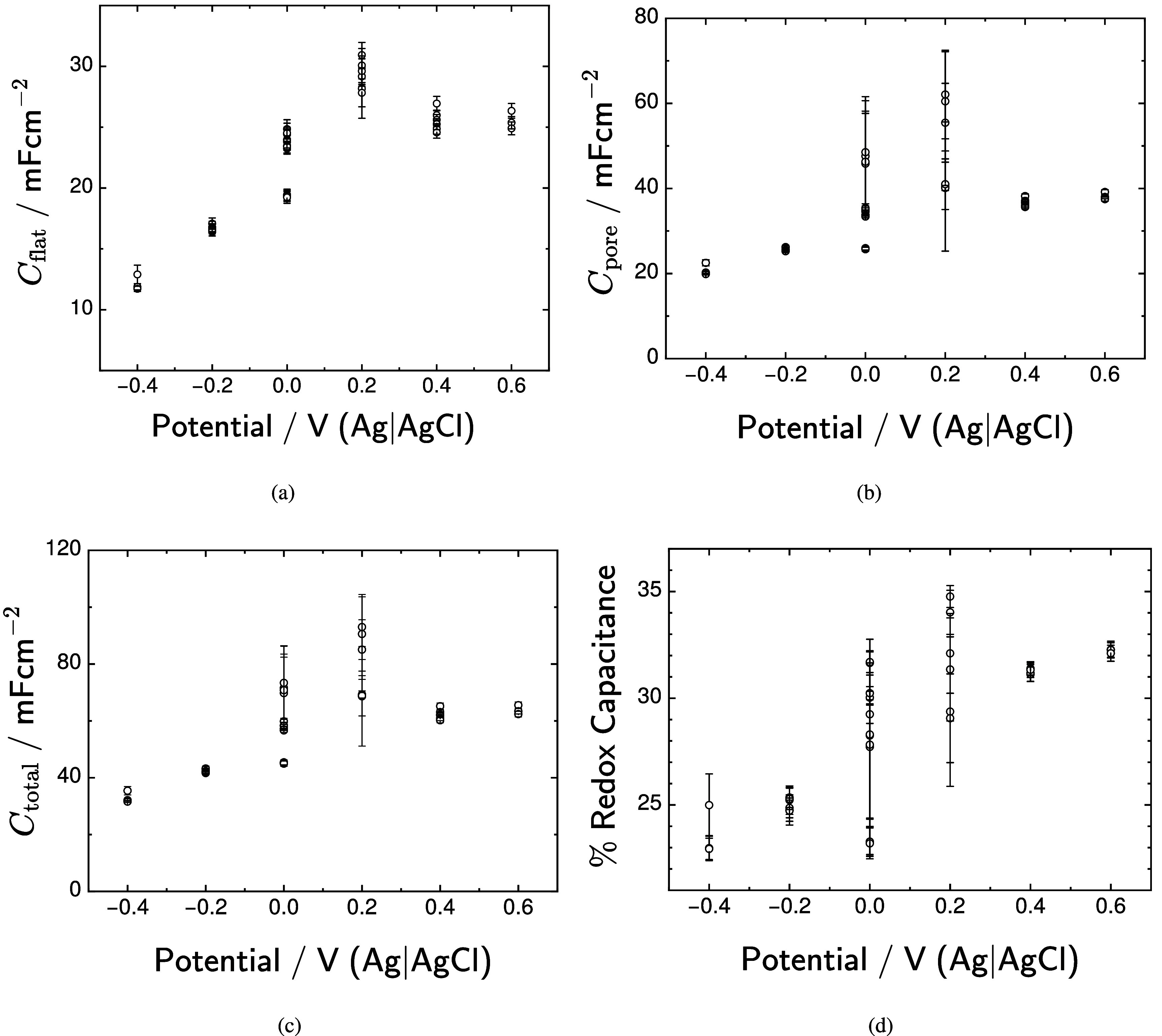
Calculated capacitances as a function of potential: (a) flat surface and pore capacitance; and (b) total capacitance. Error bars represent one standard deviation.

The calculated capacitances are presented in figure 12 as functions of potential. The total capacitance for the flat surface, presented in figure 12(a), increased with increasing potential from 12 to 30 mF cm^−2^; whereas, the total capacitance for the pore walls, shown in figure 12(b), increased from 20 to 38 mF cm^−2^. The sum of flat and pore capacitances, presented in figure 12(c), increased with increasing potential from a value of 32 mF cm^−2^ to 65 mF cm^−2^, with values reaching 93 mF cm^−2^ at 0.2 V(Ag|AgCl). The percent contribution to capacitance associated with changes in oxidation state for iridium is shown in figure 12(d) to increase with potential from 24 to 32 percent.

**Figure 13.**
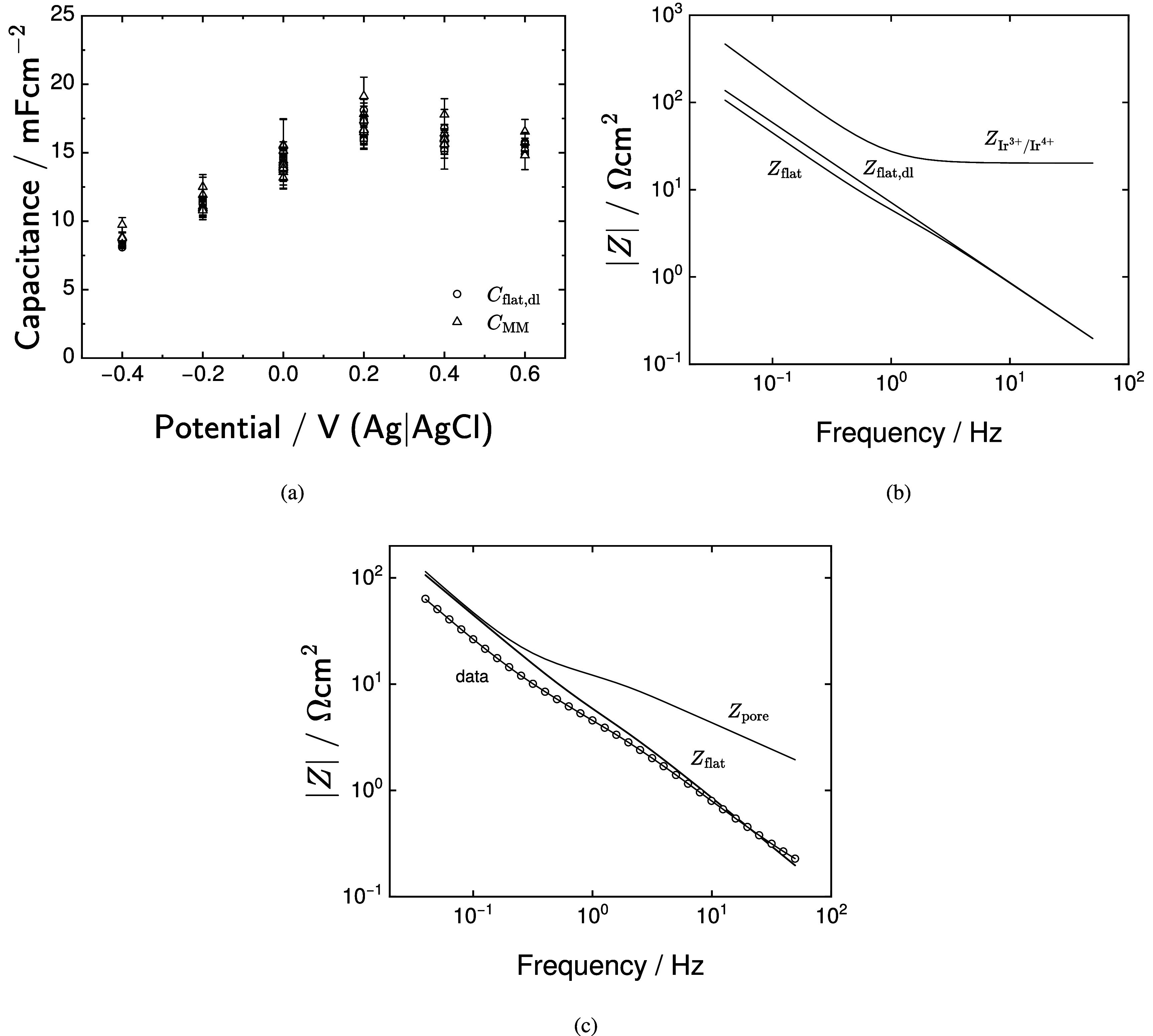
Capacitance calculated from equations (38), (43), and (44) as functions of potential: (a) total capacitance and measurement model capacitance; (b) flat capacitance and measurement model capacitance; and (c) comparison of the magnitudes of $Z_\mathrm{flat}$, $Z_\mathrm{pore}$, and the observed impedance over the frequency range of analysis. Error bars represent one standard deviation.

The capacitance values generated by the measurement model are compared with the calculated flat-surface double-layer capacitances in figure 13(a), showing that the measurement model captures the high-frequency capacitance. As shown in figure 13(b), the capacitance contribution associated with the changes in oxidation states of iridium are visible at frequencies below 1 Hz. As shown in figure 13(c), the dominant contribution to impedance at high frequency is that corresponding to the electrode surface. This result is consistent with the penetration length of the AC signal into the pores, i.e. $\lambda = 1/\sqrt{\Lambda}$. As discussed by Lasia [29], *λ* tends toward zero at large frequencies, and the AC signal cannot penetrate deeply into the pore. Thus, the dominant impedance at high frequencies is the surface impedance.

### 4.3. Assessment of capacitance from CV

**Figure 14.**
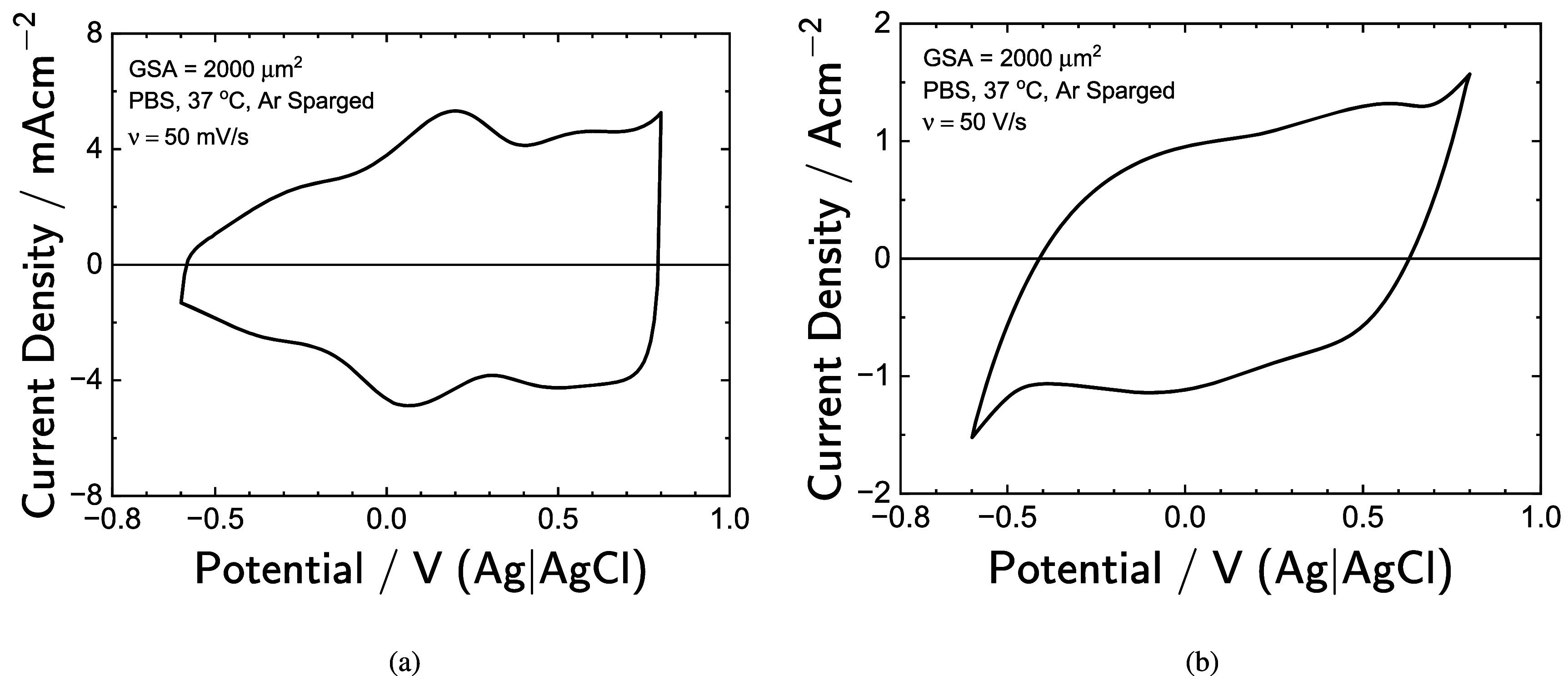
Cyclic voltammetry data the for SIROF micro-electrode: (a) a sweep rate of 50 mV s^−1^ and (b) a sweep rate of 50 V s^−1^. Note the change in current scale: mA cm^−2^ in panel (a) and A cm^−2^ in panel (b).

CV results for the SIROF micro-electrode are shown in figure 14 for sweep rates of 50 mV s^−1^ and 50 V s^−1^. The applied potential range for the sweeps was −0.6 to 0.8 V(Ag|AgCl). The range of current densities observed was an order of magnitude larger for the sweep rate of 50 V s^−1^ than for the sweep rate of 50 mV s^−1^. The cathodic charge storage capacity of the SIROF micro-electrode was calculated from the cyclic voltammograms, i.e.

\begin{equation*} q = -\int \mathrm{i}\left(t\right)\mathrm{d}t\end{equation*}

for the region where current density is negative. The parameter *q* in equation (45) is charge density in units of C cm^−2^, and *i*(*t*) is the current density in A cm^−2^. The cyclic voltammograms are presented as current density as a function of applied potential, but may be converted to current density as a function of time using the corresponding sweep rate. The charge-storage capacity of SIROF was found to be 25.6 mC cm^−2^ for a sweep rate of 50 V s^−1^ and 98.2 mC cm^−2^ for a sweep rate of 50 mV s^−1^. These values are on the order of the charge density of 107 mC cm^−2^ developed for the maximum contribution of the Ir^3+^/Ir^4+^ transition in equation (27).

The charge-storage capacities extracted from CV may be expressed in terms of capacitance following

\begin{equation*} C = \frac{q}{\Delta V}\end{equation*}

allowing comparison to capacitance values extracted from EIS. The term Δ*V* represents the range of interfacial potential, which may be calculated from applied potential

\begin{equation*} \Delta V = V_\mathrm{max}-V_\mathrm{min} -\left(i_\mathrm{max}-i_\mathrm{min}\right) R_\mathrm{e}\end{equation*}

where the ohmic resistance is that obtained from impedance spectroscopy. For the smaller sweep rate of 50 mV s^−1^, the ohmic potential drop was 0.66 mV, which is negligible when compared to potential differences on the order of 1 V. For the larger sweep rate of 50 V s^−1^, however, the ohmic potential drop correction was 0.17 V. The potential range used included all potentials for which the current was negative. Therefore, Δ*V* for the 50 V s^−1^ sweep rate was 1.06 V, ranging from −0.43 to 0.63 V(Ag|AgCl). The capacitance extracted from CV data was 70.1 mF cm^−2^ for a sweep rate of 50 mV s^−1^ and 24.2 mF cm^−2^ for a sweep rate of 50 V s^−1^.

As shown in equation (46) and for $\Delta V = 1$ V, the maximum contribution of the Ir^3+^/Ir^4+^ transition to capacity may be estimated to be *C* = 107 mF cm^−2^. The impedance analysis suggests a capacitance associated with the IrO_2_ between 10 and 20 mF cm^−2^, a value that indicates that between 10 and 20 percent of the iridium in the film contributes to the electrode capacitance.

Even the larger sweep rate of 50 V s^−1^ corresponded to a frequency of 17.5 Hz, which is low when compared to the frequency range used for impedance spectroscopy. Thus, the capacitance extracted from CV should include contributions from both the pore walls and the electrode surface. The value obtained for a sweep rate of 50 V s^−1^ was 24.2 mF cm^−2^, which is slightly lower than the range of capacitances 32–93 $\mathrm{mF}\,\mathrm{cm}^{-2}$ obtained from impedance measurements. The capacitance value obtained for a sweep rate of 50 mV s^−1^ was 70.1 mF cm^−2^, consistent with the largest values obtained from impedance spectroscopy.

## 5. Conclusions

The present work describes the development of an interpretation model based on the proposed reactions and physical structure for the impedance of SIROF micro-electrodes in phosphate-buffered saline. The success of the analysis was predicated on the removal of frequencies that were influenced by an ohmic impedance. The regression was weighted in accordance to an experimentally-measured stochastic error structure. The twelve sets of replicate impedance spectra each had a unique stochastic error structure used to weight their corresponding regression analyses. The proposed model comprised a parallel combination of a flat surface and porous surface impedances that accounted for double-layer and iridium contributions to capacitance. The model parameters extracted were found to be strong functions of potential.

The capacitance calculated from the constant-phase-element parameters under assumption of a normal distribution of time constants produced a non-physical film thickness. Therefore, a surface distribution of time constants was assumed to calculate the double-layer capacitances. The total capacitance included the contribution of double-layer and iridium oxidation states. The capacitance of both the porous surface and flat surface components depended on potential and reached a maximum between 0.2 and 0.4 V(Ag|AgCl). The comparison of the calculated capacitances to the measurement model capacitances showed that the measurement model was in agreement with the double-layer capacitance on the flat surface, but did not include the capacitance associated with the oxidation state of iridium. This result is due to the observation that the overall impedance was dominated by the flat surface double-layer component at high frequency.

The capacitance obtained in the present work includes the influence of the $\mathrm{Ir}^{3+}/\mathrm{Ir}^{4+}$ oxidation/reduction reactions. The observation that the steady-state current was equal to zero for all potentials suggests that the experiments were performed within the water window and that sufficient time was provided to allow equilibration of the $\mathrm{Ir}^{3+}/\mathrm{Ir}^{4+}$ oxidation/reduction reactions. A small peak observed in the CV for the smaller sweep rate may be attributed to the $\mathrm{Ir}^{3+}/\mathrm{Ir}^{4+}$ oxidation/reduction reactions.

The information gained from *in-vitro* experiments can guide interpretation of the impedance response for similar electrodes conducted in animal or human studies. While the impedance conducted *in vivo* is certainly influenced by the different environment, *in-vitro* studies facilitate interpretation of electrode–electrolyte properties, which comprise a component of impedance measurements performed *in vivo*.

The present work demonstrates a philosophy of interpretation modeling that accounts for both the physics and chemistry of the system and the error structure of the measurements. It is common for authors reporting the properties of electrodes used in neuromodulation applications to include EIS measurements. The depth of interpretation of EIS data is often modest, and the checking and handling of measurement error absent. The methodology described in the present work is generally applicable to all electrodes used in electrical-stimulation-based neuromodulation and will provide a framework for understanding *in-vivo* electrode behavior and informing the optimization of stimulation electrode coatings. As such, the present work has broad application to the neural engineering field.

## Data Availability

The data that support the findings of this study will be openly available following an embargo at the following URL/DOI: https://dandiarchive.org/dandiset/.
